# Does deep cerebral venous engorgement contribute to non-hydrocephalic pineal cysts becoming symptomatic? Some missing links

**DOI:** 10.1093/braincomms/fcad096

**Published:** 2023-03-29

**Authors:** Thomas Santarius, John D Pickard

**Affiliations:** Department of Clinical Neurosciences, University of Cambridge, Addenbrooke’s Hospital, Cambridge CB2 0QQ, UK; Department of Clinical Neurosciences, University of Cambridge, Addenbrooke’s Hospital, Cambridge CB2 0QQ, UK

## Abstract

This scientific commentary refers to ‘Physiological alterations of pineal recess crowding in symptomatic non-hydrocephalic pineal cysts’ by Eide *et al*. (https://doi.org/10.1093/braincomms/fcad078).

**This scientific commentary refers to ‘Physiological alterations of pineal recess crowding in symptomatic non-hydrocephalic pineal cysts’ by Eide *et al*. (https://doi.org/10.1093/braincomms/fcad078).**

## The clinical conundrum

Benign cysts of the pineal gland (PCs) are common (0.5–5% of MRI scans; 20% of autopsies). Most are small (<10 mm), remain stable or decrease in size over time and, when asymptomatic, simply require patients to be reassured. In contrast, large PCs may compress the tectum and aqueduct of Sylvius resulting in symptomatic hydrocephalus and/or Parinaud’s syndrome. The management of symptomatic PCs with hydrocephalus is well established—endoscopic third ventriculostomy or shunt to decompress the hydrocephalus and endoscopic biopsy/fenestration to confirm its benign nature and decompress the cyst.^1^

However, considerable controversy surrounds the diagnosis, pathophysiology and management of non-hydrocephalic PCs that are symptomatic (nhSPCs). Symptoms may appear to be rather non-specific and the demonstration of a PC on CT/MRI dismissed as incidental. Symptoms may include paroxysmal and chronic headaches, visual disturbances/gaze paresis, nausea, sleep disorders, brain fog, fatigue, dizziness, gait instability, depression, cognitive impairment, transient neurological deficits, episodic unconsciousness and seizures in the presence of a PC and absence of ventriculomegaly. Patients may be profoundly miserable with greatly impaired quality of life.

A recent systematic review of surgical intervention found that, in highly selected cases with severe symptoms impacting on quality of life, improvement rate was 93%. Although surgery in the pineal region is technically challenging, in skilled hands, the overall complication rate was 17% with long-term consequences in 3.6%.^2^ A plea was made for prospective studies with patient-reported and objective outcome assessment to provide a higher level of evidence.

What mechanisms might underlie the effects of pineal cysts both locally and on the brain as a whole? Pineal cysts are in close proximity to and may compress key structures affecting vision (superior colliculi, upper tectum, tegmentum anteriorly to the aqueduct, posterior and habenular commissures), hearing and balance (inferior colliculi and lateral lemniscus), and concentration, memory and emotion (habenula, thalamus, and tegmentum) as well as more widely distributed functional networks drained by the deep venous system (including the thalami, basal ganglia and its projection fibres, and mesial temporal lobes).^1^ When present, episodic positional headaches, paroxysmal headaches associated with bilateral visual blurring or diplopia, and unexplained episodic loss of consciousness are suggestive of intermittent CSF obstruction and/or pressure on the posterior midbrain even if ventriculomegaly is not present.^3^

## Hypothesis

Eide *et al*.^4,5^ have previously proposed the intriguing hypothesis that, in some nhSPCs, crowding of the pineal recess may impair the venous runoff from the internal cerebral veins (ICVs) resulting in deep cerebral venous hypertension thereby, they postulate, increasing interstitial water content within adjacent structures including the thalamus.

Their hypothesis comes at a time of growing awareness of the potential role of cerebral venous congestion in other conditions, a concept that has led to new therapeutic opportunities. ^6^

## The evidence

###  

#### The symptoms

Many symptoms that patients with nhSPCs complain of overlap with those of cerebral venous thrombosis, idiopathic intracranial hypertension (IIH) and markedly asymmetrical cerebral venous outflow, all conditions with indisputable venous components. However, there are few detailed studies of the headache, cognitive, visual, hearing, balance and gait aspects in relation to the structures and systems that may be involved.

#### What defines a crowded pineal recess?

The Oslo group found that the anteroposterior diameter of the cyst or tectal compression and stenosis of the Sylvian aqueduct were not associated with symptom severity. However, MRI-based biomarkers of a crowded pineal recess [thalamic–splenium–cyst (TSC) ratio and thalamic apparent diffusion coefficient (ADC) ratio—see below] were associated with severity of symptoms, intracranial pressure (ICP) scores and outcome after surgery. ^4^

#### Evidence for cerebral venous engorgement

Ideally, direct evidence for venous engorgement would be based on quantitative CT/MR venography, regional cerebral blood volume estimation (preferably both arterial and venous) and, although probably not feasible, retrograde cerebral venography with pressure gradient measurements. What were the dimensions of the postulated ICV narrowing around the PC? Were there any congenital variations or collateral changes in central venous drainage or areas of stenosis remote from the pineal cyst region? Unfortunately, such direct evidence has not yet been reported. Instead, blood flow velocity (FV) in the ICVs, CSF FV in the aqueduct and intrathecal gadolinium distribution (‘glymphatic function’) have been assessed in relation to pineal recess crowding in 35 patients with nhSPCs.^5^

#### Is ICP raised in patients with nhSPCs compared with those with chronic daily headache?

Venous congestion might be expected to raise ICP. However, mean ICP was not raised in either group.^4^ Wave amplitude was greater by 1.3 mmHg which suggests that any venous congestion is regional only and/or venous collaterals have opened up.

#### Interstitial oedema

Obstruction of venous vessels increases venous pressure, reduces capillary perfusion and increases local cerebral blood volume, compensated for by recruitment of collateral vessels. Where compensation fails, both local cytotoxic and vasogenic oedema may occur.

However, the Oslo group propose that the hydrostatic pressure gradient created by venous back pressure induces interstitial oedema. Conventionally, interstitial cerebral oedema refers to transependymal oedema in the periventricular white matter (WM), not grey matter (GM), caused by hydrocephalus. FLAIR is the most sensitive MRI sequence for detection of interstitial oedema but has not been reported to show such changes in the WM or GM around the ventricles or pineal recess in nhSPCs (or in IIH).

For logistical reasons, the authors measured ADCs in different regions of interest, not in absolute terms but as ratios with central hemispheric WM as the reference ROI. Whether central WM is truly independent of venous back pressure from the ICVs is debatable—venous territories are less well defined than arterial.

Only the thalamic-central WM ADC ratio showed any significant changes with symptom severity (0.99 versus 0.96) and ICP pulsatile pressure (1.02 versus 0.98). Importantly, thalamic-central WM ADC ratio did not change after cyst removal. Going forwards, more advanced MR diffusion tensor imaging (DTI) techniques will provide a more comprehensive understanding of water movement in structures around and distant from the pineal recess. ^7^

#### Aqueductal flow

CSF reflux through the aqueduct was demonstrated with PC MRI (25% of cases) and intrathecal gadolinium MRI (40% of cases).

#### FV measurements

FV was measured with PC MRI in the ICVs proximal to (upstream) and at the cyst, not downstream to the cyst before the ICVs enter the vein of Galen. There were no significant differences between surgical and conservative groups in per cent changes in ICV FVs at the cyst. In the combined groups, there was a small increase in ICV FV at the cyst of the order of 0.5 cm/s which was less as the cyst increased in size (TSC ratio). Without knowledge of the dimensions of the ICVs and venous anatomy, it is difficult to interpret such FV changes.

It is instructive to compare these small changes with the much larger changes in trans-stenotic blood FVs (4D flow MRI: upstream—downstream, not at the stenosis) in relation to the cerebral venous gradient (retrograde cerebral venography with manometry) across transverse sinus stenoses (IIH and pulsatile tinnitus).^8^ There were no differences between FVs upstream between patients and controls. However, the average change in FV downstream in the transverse sinus in controls was 4.2 and 21.5 cm/s in the patients. For every increase of 10 cm/s in average FV, the cerebral venous gradient increased by 2.0 mmHg. 4D MRI displayed rather dramatic flow jets downstream in the patients. Unlike arterial pressure gradients, apparently small cerebral venous gradients may still be haemodynamically significant.

#### Intrathecal gadolinium MR enhancement

The authors use the term ‘glymphatic enrichment’ to refer to CSF-mediated molecular enhancement in brain along extravascular pathways, specifically CSF influx along penetrating cortical arteries. They used intrathecal gadolinium MRI which lacks the spatial (1 mm) and temporal (hours) resolution required to demonstrate the postulated microscopic glymphatic machinery.^9^ There is considerable debate about the exit route for large molecules.^10^

Despite CSF reflux through the aqueduct in some patients, the tracer arrived in the cisterna magna and pineal recess and then the cortical subarachnoid space with much greater enrichment and more rapidly than into the ventricular system, using orbital fat as the reference. Tracer enhancement within the cerebral cortex was much greater than in subcortical WM and thalamus; all decreased with increasing crowding of the pineal recess. Curiously, this was only significant for the thalamic ADC ratio component, not for the TSC ratio. Notably, in the surgically treated patients who differed by severity of symptoms from the conservatively managed group, tracer enrichment was modestly reduced in the cerebral cortex and subcortical WM. This contrasts with the increased tracer enrichment reported by the Oslo group in IIH patients.

## Conclusions

There is growing awareness that there is a small cohort of patients with very disabling nhSPCs who have had positive and sustained outcomes after surgical decompression. A higher level of evidence is now required. This will require a multicentre registry to foster patient engagement, develop patient-reported and objective outcome assessments, facilitate exploratory pathophysiological studies and design a multicentre RCT.

The Oslo group are to be congratulated on their thought-provoking hypothesis that will stimulate much further multidisciplinary study and independent verification for which the tools are now available.

## Data Availability

No new data had been provided or analysed in this Commentary.
